# The Complex Interaction Between Sleep-Related Information, Misinformation, and Sleep Health: Call for Comprehensive Research on Sleep Infodemiology and Infoveillance

**DOI:** 10.2196/57748

**Published:** 2024-12-13

**Authors:** Nicola Luigi Bragazzi, Sergio Garbarino

**Affiliations:** 1 Human Nutrition Unit, Department of Food and Drugs University of Parma Parma Italy; 2 Department of Neuroscience, Rehabilitation, Ophthalmology, Genetics and Maternal/Child Sciences University of Genoa Genoa Italy

**Keywords:** sleep health, sleep-related clinical public health, sleep information, health information, infodemiology, infoveillance, social media, myth, misconception, circadian, chronobiology, insomnia, eHealth, digital health, public health informatics, sleep data, health data, well-being, patient information, lifestyle

## Abstract

The complex interplay between sleep-related information—both accurate and misleading—and its impact on clinical public health is an emerging area of concern. Lack of awareness of the importance of sleep, and inadequate information related to sleep, combined with misinformation about sleep, disseminated through social media, nonexpert advice, commercial interests, and other sources, can distort individuals’ understanding of healthy sleep practices. Such misinformation can lead to the adoption of unhealthy sleep behaviors, reducing sleep quality and exacerbating sleep disorders. Simultaneously, poor sleep itself impairs critical cognitive functions, such as memory consolidation, emotional regulation, and decision-making. These impairments can heighten individuals’ vulnerability to misinformation, creating a vicious cycle that further entrenches poor sleep habits and unhealthy behaviors. Sleep deprivation is known to reduce the ability to critically evaluate information, increase suggestibility, and enhance emotional reactivity, making individuals more prone to accepting persuasive but inaccurate information. This cycle of misinformation and poor sleep creates a clinical public health issue that goes beyond individual well-being, influencing occupational performance, societal productivity, and even broader clinical public health decision-making. The effects are felt across various sectors, from health care systems burdened by sleep-related issues to workplaces impacted by decreased productivity due to sleep deficiencies. The need for comprehensive clinical public health initiatives to combat this cycle is critical. These efforts must promote sleep literacy, increase awareness of sleep’s role in cognitive resilience, and correct widespread sleep myths. Digital tools and technologies, such as sleep-tracking devices and artificial intelligence–powered apps, can play a role in educating the public and enhancing the accessibility of accurate, evidence-based sleep information. However, these tools must be carefully designed to avoid the spread of misinformation through algorithmic biases. Furthermore, research into the cognitive impacts of sleep deprivation should be leveraged to develop strategies that enhance societal resilience against misinformation. Sleep infodemiology and infoveillance, which involve tracking and analyzing the distribution of sleep-related information across digital platforms, offer valuable methodologies for identifying and addressing the spread of misinformation in real time. Addressing this issue requires a multidisciplinary approach, involving collaboration between sleep scientists, health care providers, educators, policy makers, and digital platform regulators. By promoting healthy sleep practices and debunking myths, it is possible to disrupt the feedback loop between poor sleep and misinformation, leading to improved individual health, better decision-making, and stronger societal outcomes.

## The Role of Sleep at the Individual, Occupational, and Public Level

Ensuring a sufficient amount of high-quality, restorative sleep (“good sleep”) is fundamentally critical for the health and well-being of individuals and society at large [1]. For individuals, the benefits of sound sleep are manifold: it underpins optimal physical health, fortifies mental and emotional resilience, sharpens cognitive functions, and fosters a sense of well-being [2-6]. Moreover, it plays a protective role by diminishing the likelihood of developing chronic health conditions such as obesity, diabetes, cardiovascular diseases, neurodegenerative disorders, and malignancies, which are often exacerbated by poor sleep patterns [7-10].

On a broader scale, the implications of adequate sleep extend far beyond individual health, influencing various facets of clinical public health and societal function. Quality sleep contributes to the vigor of the economy by enhancing productivity, reducing workplace accidents, and fostering a more dynamic and engaged workforce. In terms of public safety, well-rested individuals are less likely to be involved in accidents, including those related to vehicle operation, thereby safeguarding communities [11-13].

Furthermore, addressing sleep deficiencies and disorders can have a significant impact on health care systems. By reducing the prevalence of sleep-related issues, we can alleviate the immense burden they place on health care resources, from direct medical costs to indirect expenses related to lost productivity and diminished quality of life [11-13].

## Improving Healthy Sleep Habits

Strategic interventions aimed at improving sleep health can, therefore, offer dual benefits: they can improve the overall health and quality of life for individuals while simultaneously reducing economic strains linked to health care expenditures and productivity losses. In essence, promoting better sleep practices and addressing sleep disorders is not just a matter of individual health but an occupational and clinical public health imperative with wide-reaching implications for societal well-being and economic stability [14,15].

Therefore, advocating for healthy sleep habits and actively addressing sleep disorders are crucial strategies that lead to significant improvements in individual and societal well-being. These efforts enhance productivity, strengthen community bonds, and contribute to a more harmonious social environment. By implementing such measures, the benefits are twofold, positively impacting individuals by improving their health and quality of life, and communities by fostering a more productive and cohesive population [14,15].

Promoting good sleep involves a combination of measures and interventions, including sleep hygiene practices, medications, and behavioral interventions. Sleep hygiene is crucial for achieving restful sleep and for overall well-being. Maintaining a consistent sleep schedule helps regulate the body’s internal clock. Creating a restful sleep environment is essential as well. Additionally, physical activity and dietary considerations are important. For individuals who struggle with sleep despite good sleep hygiene, medications may be prescribed, including over-the-counter drugs, benzodiazepines, non-benzodiazepine hypnotics (Z-drugs), melatonin supplements, or more recently approved drugs such as dual orexin receptor antagonists. Behavioral therapies, such as cognitive behavioral therapy for insomnia, can effectively treat insomnia and other sleep disorders.

The role of modern technology provides new opportunities for promoting good sleep. Innovative digital tools, ranging from engaging websites and interactive platforms to sophisticated sleep-tracking devices, mobile apps, chatbots, and other artificial intelligence (AI)–enhanced digital assistants, are at the forefront of promoting sleep health awareness and education. These technologies not only offer personalized insights into sleep patterns but also provide actionable guidance to improve sleep quality. By leveraging digital solutions, we can make significant strides in making sleep health information more accessible and engaging for a wide audience, thereby encouraging widespread adoption of healthier sleep practices [16-18].

The concerted effort to promote better sleep through traditional interventions such as medication use, therapy, and sleep hygiene enhanced by modern digital tools (eg, digital cognitive behavioral therapy for insomnia) holds the promise of creating a ripple effect of benefits across individual and community levels. This holistic approach to sleep health has the potential to transform societal well-being, productivity, and social cohesion in profound ways [19,20].

If comprehensively organized and combined with professional training and enhanced surveillance, these efforts can mitigate the clinical public health burden posed by chronic sleep loss and sleep disorders, which is magnified and compounded by the widespread lack of awareness among the general population, health care professionals, and policy makers [21].

Sleep is, indeed, often undervalued by individuals and society, seen as nonproductive, optional, or even stigmatized, with phrases like “time is money” reinforcing this view. Modern lifestyles driven by work schedules and globalization disrupt natural sleep patterns, leading to increased sleep deprivation and related health issues. People with sleep disorders often face stigma [22-24], which complicates their ability to seek help, as sleep is perceived as an “asocial” activity. This stigma, combined with a lack of clinical public health education on sleep, discourages individuals from discussing sleep problems with health care providers, despite the serious consequences of untreated sleep disorders [21].

## Seeking for Sleep-Related Information

For individuals grappling with sleep disorders like insomnia, the search for dependable solutions often leads to a complex information landscape. This search, driven by a need for better sleep quality, occurs within a digital ecosystem where credible medical guidance is often mixed with a variety of alternative sources, such as anecdotal recommendations, commercial promotions, and social media content. These individuals are confronted with a vast array of information, ranging from scientifically validated approaches to widespread misinformation.

Research highlights that a significant portion of people with sleep disorders do not actively seek help. For instance, a 2017 survey of Austrians found that while chronic insomnia affected 7.8% of respondents, only about half sought medical assistance [25]. This low level of help-seeking behavior is not unique to Austria; similar trends have been reported in other countries [26-28].

This discrepancy may be attributed to a range of factors, including individual (medical and psychological) and societal (informational) drivers. Clinical factors encompass the underlying health status of individuals, which may complicate diagnosis and treatment, impacting health-seeking behaviors. Psychological drivers, such as stigma, fear of diagnosis, and a reluctance to acknowledge the severity of the condition, also play a significant role, as previously mentioned. Informational barriers include the overwhelming volume of available information and confusion about treatment options. Additionally, socioeconomic factors like lack of access to health care and financial constraints [29,30], as well as cultural attitudes towards sleep disorders, further contribute to the low rates of help-seeking behavior.

As such, for individuals with insomnia and other sleep issues, navigating the information environment can be daunting, particularly as the disorder is often accompanied by anxiety, which exacerbates reassurance-seeking behaviors, such as excessive web searching for health-related information. Such behaviors tend to increase during clinical public health crises, like the COVID-19 pandemic, where misinformation is rampant [31]. The link between anxiety and sleep disturbances creates a vicious cycle where exposure to misleading information can heighten both anxiety and sleep problems [32].

Commercial influences also play a significant role in shaping the sleep health landscape. The wellness industry frequently promotes unproven treatments, such as supplements and devices, capitalizing on the vulnerability of individuals desperate for solutions [33,34]. This commercialization fosters the spread of misinformation and may lead people to adopt ineffective or even harmful practices [35,36].

Understanding how people seek and interpret sleep-related information is crucial for clinical public health initiatives. Effective interventions should go beyond merely providing accurate information or debunking misinformation—they must also empower individuals to critically assess the content they encounter and make informed decisions about their sleep health [37,38]. By addressing the clinical, psychological, informational, and commercial factors that influence help-seeking behavior, clinical public health strategies can better support individuals in adopting healthier sleep practices while reducing the spread of misinformation [39-42].

## The Impact of Sleep Misinformation on Sleep Lifestyles

Misinformation regarding sleep can significantly distort public perceptions and behaviors towards sleep, leading to unhealthy sleep lifestyles and poor-quality sleep [43-45]. The prevalence of myths and misconceptions about sleep, such as the effectiveness of sleep aids, the necessity of 8 hours of sleep for everyone, or the undervaluation of sleep’s impact on health, can misguide individuals in their sleep practices. Misinformation can emanate from various sources, including social media, nonexpert advice, and misleading marketing from sleep-related products, which often prioritize profit over factual accuracy. The perpetuation of sleep myths can have tangible consequences on sleep quality and overall health. Several sleep myths can, indeed, significantly impact sleep problems such as insomnia, low sleep efficiency, and poor sleep quality. For instance, the myth that “if you can get it, more sleep is always better” can be particularly detrimental for individuals with insomnia. Trying to compensate for lack of sleep by staying in bed longer can lead to further sleep fragmentation and more time lying awake struggling to stay asleep. Conversely, restriction of time in bed is one of the most effective behavioral treatments for insomnia. Another harmful myth is the belief that “if you are having difficulties sleeping at night, taking a nap in the afternoon is a good way to get adequate sleep.” Napping is discouraged among those with insomnia as it may reduce homeostatic sleep drive (process S) and perpetuate nighttime insomnia. Similarly, the misconception that one can “catch up” on lost sleep during weekends can lead to erratic sleep patterns that disrupt the body’s natural circadian rhythm and other biological rhythms, exacerbating sleep problems. Low sleep efficiency can be negatively impacted by the myth that “lying in bed with your eyes closed is almost as good as sleeping.” This belief is harmful as endocrine, cardiovascular, metabolic, and cognitive functions are markedly different during wakefulness than during nonrapid eye movement sleep. Believing this myth may lead individuals to spend excessive time in bed without actually sleeping, thereby reducing overall sleep efficiency. Poor sleep quality is also affected by myths such as “alcohol before bed will improve your sleep.” While alcohol may reduce sleep latency, it subsequently causes sleep disturbances in the second half of the night, increasing slow-wave sleep nonrapid eye movement, delaying the onset of rapid eye movement sleep, and worsening overall sleep quality. It also exacerbates sleep apnea symptoms, further degrading sleep quality. Another myth affecting sleep quality is the belief that “for sleeping, it is better to have a warmer bedroom than a cooler bedroom”. A warm environment is associated with poor sleep, whereas studies have shown that a cooler bedroom is preferable for good sleep quantity and quality. These examples highlight how misconceptions about sleep can adversely affect individuals’ sleep patterns and overall health [44,45].

The internet, platforms like YouTube, TikTok, and other web-based channels serve as double-edged swords in the quest for health literacy. On one hand, they offer unprecedented access to information, potentially enhancing public understanding and awareness of health issues, including sleep disorders and insomnia. On the other hand, the most popular and visible content on these platforms is not always accurate and can lead to the widespread dissemination of misinformation, as shown by a recently published study by Robbins et al [46]. This study underscores several critical points, including the role of commercial biases, finding that a significant majority (67%) of the popular YouTube videos on sleep and insomnia exhibited signs of commercial bias. This implies that these videos might prioritize advertisements or promotions of sleep-related products (from sleep aids to bedding items) or endorsements of sleep-related services (such as sleep therapy programs) over factual or beneficial health information (like their efficacy), potentially misleading viewers for commercial gains. Moreover, misinformation was more commonly found in popular videos, which also happened to have significantly higher viewership (averaging 8.2 million views) compared with expert-led videos (with around 0.3 million views). This discrepancy highlights a concerning trend where misleading content has a broader reach and impact compared with accurate, expert-driven information. This discrepancy can be explained by taking into account that the most viewed YouTube videos on sleep and insomnia tend to be designed to appeal to shorter attention spans, featuring engaging content, high visual quality, and relatability. While these characteristics can make information more accessible and engaging, they also raise concerns when used to propagate misleading or biased information.

Social media platforms, search engines, and other digital ecosystems use sophisticated algorithms to personalize content for users. While this can enhance user experience, it also has significant implications for the spread of misinformation, conspiracies, and for-profit posts, particularly in the context of sleep health. Algorithms are designed to maximize user engagement by prioritizing content that aligns with users’ interests and viewing habits. This often leads to an echo chamber effect, where individuals are repeatedly exposed to similar types of content, reinforcing their existing beliefs and biases. In the context of sleep health, this can mean that misinformation, such as myths about sleep aids or the misinterpretation of sleep science, is disproportionately amplified. A user who watches or engages with a video promoting an unproven sleep remedy is likely to see more similar content, perpetuating misinformation and potentially leading to harmful sleep practices. Conspiracy theories often thrive in algorithm-driven environments due to their engaging and sensational nature. Algorithms that prioritize engagement can inadvertently promote conspiracy theories related to sleep, such as the notion that certain sleep medications are part of a larger pharmaceutical conspiracy. These theories can spread rapidly as algorithms push content that generates high levels of interaction, irrespective of its veracity. This not only misguides individuals but can also erode trust in credible sleep health information and professionals. Many platforms allow for-profit entities to promote their products through paid posts. In the realm of sleep health, this often includes supplements, sleep aids, and devices that may not be backed by scientific evidence, as previously mentioned. Algorithms that optimize for advertising revenue can prioritize these paid posts over more accurate, noncommercial content. As a result, users are frequently exposed to and potentially influenced by misleading information that prioritizes profit over health.

Addressing the pervasive issue of sleep-related misinformation is urgent and requires a collaborative research effort to develop effective strategies. Researchers can help improve vetting processes for health-related content, and promote credible, expert-led information sources. Ensuring the reliability and quality of health information in the digital age presents, indeed, significant challenges and involves adopting a critical approach to evaluating web-based health content, emphasizing the importance of evidence-based information, and rigorously identifying biases and inaccuracies. By advancing these initiatives, researchers can support public health efforts and foster a more informed and resilient society [47-49].

## The Vulnerability to Misinformation Due to Poor Sleep

On the flip side, poor sleep itself can render individuals more susceptible to misinformation, creating a vicious cycle that further entrenches unhealthy sleep lifestyles. Sleep is crucial for various cognitive functions, including memory consolidation, emotional regulation, and critical thinking. Lack of adequate sleep can impair these cognitive processes, making individuals more prone to cognitive biases and less capable of discerning credible information from misinformation [50]. Recent research highlighted that sleep deprivation can lead to increased suggestibility, reduced ability to process information critically, and heightened emotional reactivity [51]. These poor sleep-induced cognitive impairments can make individuals more susceptible to persuasive misinformation, especially when it appeals to emotional biases or confirms preexisting beliefs. In the era of information overload and sophisticated misinformation, the ability to critically evaluate information is paramount, and sleep deprivation undermines this critical capacity.

When people observe an event and later encounter incorrect information about it, they often blend this inaccurate information into their memory of the event, a phenomenon referred to as the “misinformation effect.” A study by Calvillo et al [52] delved into how sleep influences this effect. A total of 177 participants were involved; they observed 2 events and were then presented with misleading information either immediately, 12 hours later on the same day, 12 hours later on the following day, or 24 hours after the events. Subsequently, they underwent a recognition test. The findings indicated that all participant groups were susceptible to the misinformation effect, with the effect being more pronounced in those who had a sleep period before the test. Analysis using signal detection theory showed that sleep reduced the ability to discern between accurate and misleading information. These outcomes imply that sleep may heighten vulnerability to the misinformation effect, possibly because sleep tends to consolidate the general essence of the original events in memory or enhances the assimilation of the misleading information presented after the event.

However, the relationship between sleep and the development of incorrect memories/misinformation effect is not clear-cut, with some studies suggesting that sleep leads to an increase in incorrect recollections, while others indicate a reduction in false recognitions. In a study by Day and Fenn [53], participants watched a video of a house burglary, were given misleading information about the video, and then had their memory tested. The memory test took place after a 12-hour period that included either a period of sleep or wakefulness. The point at which the misleading information was provided varied: half of the participants received it immediately after viewing the video (before the sleep or wake period), and the other half received it after the 12-hour interval (post sleep or wakefulness). A significant effect of the experimental condition on accurate memory recall could be detected, with those in the sleep condition showing better accurate memory than those who remained awake. Regarding the creation of false memories, a significant effect based on the timing of the misleading information was observed, as well as an interaction between the experimental condition and the timing of this information. Specifically, the impact of sleep on false memories was influenced by the timing of the misinformation delivery. When misleading information was provided after the retention period, sleep tended to reduce false memories compared with wakefulness. However, if the misleading information was given before the retention period, sleep appeared to increase the likelihood of false memories. In conclusion, sleep has the dual potential to either guard against or promote the distortion of memories, contingent upon the timing of exposure to misinformation. These findings enhance the understanding of memory consolidation processes. When consolidation is focused solely on accurate memories, it fortifies those memories, making them less susceptible to inaccuracies. On the other hand, if consolidation occurs in the presence of misinformation before sleep, it might integrate this incorrect information into the memory of the actual event, thereby increasing the chance of memory distortion.

Further studies are needed to explore the timing of sleep, its quality, and its interaction in influencing the misinformation effect and memory consolidation processes. Understanding how different factors of sleep contribute to memory distortion or protection can provide deeper insights into the complexities of sleep and cognitive function, guiding future interventions aimed at reducing susceptibility to misinformation.

## The 24-Hour Society and the Era of Posttruth and Deepfakes

**Figure 1:**
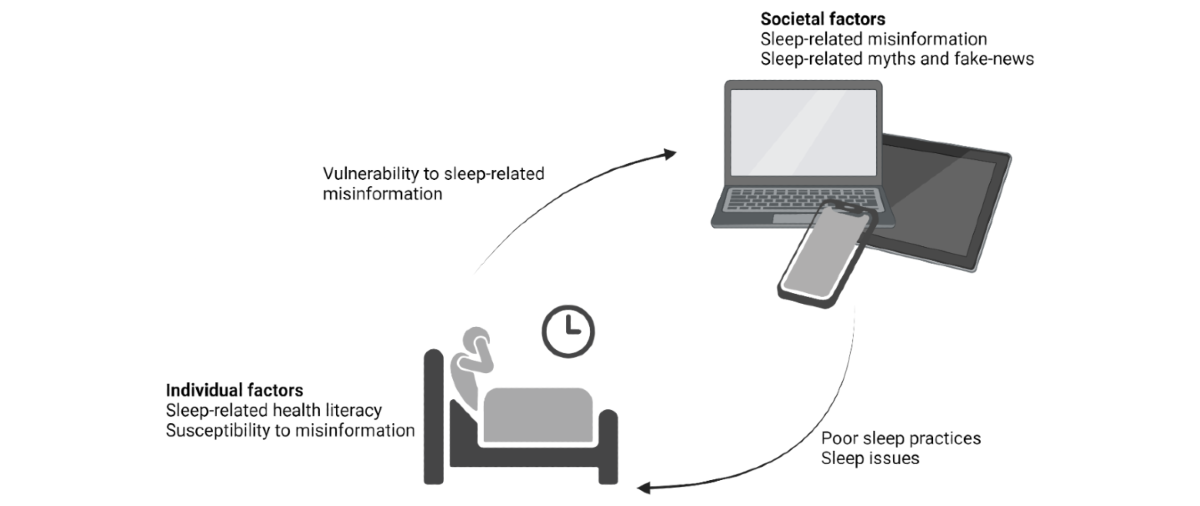
The vicious cycle of the impact of sleep-related misinformation on sleep health and poor sleep-induced vulnerability to misinformation in the 24-hour society and in the era of posttruth and deepfakes. Figure created with BioRender.com.

The reciprocal relationship between sleep-related misinformation and susceptibility to misinformation due to poor sleep or unhealthy sleep habits creates a feedback loop that can be challenging to break (Figure 1). This cycle not only affects individual health and well-being but also has broader societal implications. For instance, in the context of clinical public health, widespread sleep-related misinformation can undermine efforts to address sleep disorders and promote healthy sleep practices, while a population increasingly susceptible to misinformation due to poor sleep or unhealthy sleep habits can exacerbate the spread of false information, with potential consequences for public health decisions and behaviors.

In the 24-hour society, where round-the-clock connectivity and the demands of a global economy disrupt natural sleep patterns, the cycle of sleep-related misinformation and susceptibility to misinformation due to poor sleep or unhealthy sleep habits is particularly pernicious. This is further compounded by the posttruth era’s challenges, where objective facts are less influential in shaping public opinion than appeals to emotion and personal belief.

The proliferation of deepfakes (that is to say, AI-generated synthetic media that convincingly alters images and videos, posing risks to information integrity and privacy) and sophisticated misinformation can further erode trust in authoritative health advice, including guidance on sleep. As individuals navigate this complex information landscape with impaired cognitive faculties due to sleep deprivation, the discernment between fact and fiction becomes increasingly blurred. This not only hampers the individual’s ability to make informed decisions regarding their own sleep health but also undermines collective efforts to foster a well-informed public capable of critical thinking in the face of pervasive misinformation. The resulting societal impact is a society less equipped to engage with and support evidence-based clinical public health initiatives, leading to broader clinical public health challenges and diminished resilience against misinformation-driven health crises.

## The Potential Role of Sleep Infodemiology and Infoveillance

Infodemiology can be defined as “the science of distribution and determinants of information in an electronic medium, specifically the internet, or in a population, with the ultimate aim to inform public health and public policy” [54]. Infodemiology uses sophisticated algorithms and data analytics tools to sift through vast amounts of web-based data, including search engine queries, social media posts, and discussions on health forums [54-60]. This is known as “infoveillance,” which is short for information surveillance. By systematically collecting information mostly from digital sources and analyzing trends in this data, such as spikes in certain sleep-related queries or widespread sharing of particular pieces of sleep advice, researchers can identify what sleep topics are of most concern to the public and where there may be gaps or inaccuracies in their knowledge.

For example, if there is a noticeable increase in searches for “benefits of sleeping less,” it might indicate a growing misconception that less sleep is somehow advantageous, signaling a need for corrective public health messaging. Infoveillance extends these insights by providing continuous, automated monitoring of health information flow on the internet. This real-time aspect is crucial for quickly identifying and responding to emerging sleep myths or misinformation before they have a chance to become widely accepted. For instance, if a new but unfounded claim about a sleep supplement begins trending on social media, infoveillance systems can flag this trend, enabling health authorities to respond promptly with evidence-based information to counteract the misinformation. Furthermore, these methodologies can identify influential nodes within web-based networks—such as key social media influencers or websites—that propagate sleep myths. Clinical public health campaigns can then engage with these nodes directly or use their reach to disseminate accurate sleep-related information more effectively. Digital platforms, through their algorithms, often prioritize engagement over accuracy, potentially amplifying misinformation. By implementing robust content moderation, integrating fact-checking mechanisms, increasing transparency, and actively promoting accurate information, digital platforms can play a crucial role in disseminating credible health information and curbing the spread of sleep myths.

Additionally, infodemiology and infoveillance can help tailor clinical public health messages to specific demographics or communities by analyzing the types of sleep-related misinformation that are most prevalent within those groups. This targeted approach ensures that interventions are relevant and resonate with the intended audience, increasing the likelihood of their success.

In summary, by harnessing the power of big data and digital monitoring, infodemiology and infoveillance offer potent tools for disseminating high-quality sleep-related information and combating sleep-related misinformation. They provide a nuanced understanding of public perceptions and behaviors around sleep, enabling the development of more effective, timely, and targeted clinical public health responses to ensure that accurate, reliable, and accessible sleep-related information reaches those who need it most.

## A Call for Research on Sleep-Related Information, Misinformation, Sleep Infodemiology, and Infoveillance

Given the scarce and, at times, conflicting literature on the intersection of sleep-related information, misinformation, and their broader impacts, there is a pressing need for comprehensive research in this area. This call for research extends to the emerging superspecialties of sleep infodemiology and infoveillance, aiming to systematically understand and monitor the spread and effects of sleep-related information and misinformation across digital platforms and their implications for clinical public health. The complexity of sleep-related information and misinformation, compounded by the rapid evolution of digital communication technologies, necessitates, indeed, multidisciplinary research approaches. This includes collaborations among sleep scientists, psychologists, sociologists, information scientists, and technologists to explore the nuances of how sleep-related information is disseminated, interpreted, and acted upon in various contexts.

Research should specifically aim to (1) map the landscape of sleep-related information; (2) understand the drivers of sleep-related information-seeking behaviors and how “sleep-related information diets” influence individual actions; (3) construct behavioral models from a socioecological perspective; (4) explore the role of digital platforms in shaping sleep health, develop, and implement sleep health–related information interventions while evaluating their effectiveness; (5) identify gaps and risk factors in sleep health–related communication; and (6) establish metrics and analytical methods for advancing sleep infodemiology and infoveillance.

More in detail, research should catalog and analyze the various sources and types of information related to sleep available to the public, identifying key players and informational voids, including identifying the most prevalent forms of sleep-related misinformation, their origins, and the mechanisms through which they spread. This involves analyzing content across various media, including social networks, blogs, forums, and news outlets, to understand the scope and scale of sleep-related information and misinformation. Researchers should investigate the direct and indirect effects of sleep-related information on individual health behaviors and outcomes, as well as on broader clinical public health initiatives. This includes assessing the impact on sleep hygiene practices, the prevalence of sleep disorders, the prescription and consumption of sleep drugs, the use of sleep devices, and the public’s trust in health authorities and scientific evidence. Moreover, researchers should design, implement, and assess the effectiveness of interventions aimed at improving sleep health, focusing on both efficacy and user engagement, disseminating high-quality sleep-related information, and combating sleep-related misinformation. This could involve educational campaigns, digital literacy programs, and the development of tools and algorithms for detecting and countering misinformation via the web. Further, researchers should analyze how algorithms, user interfaces, and platform policies contribute to the spread of sleep-related misinformation and identify opportunities for collaboration with technology companies to promote accurate information and curb misinformation.

Other research areas of sleep infodemiology and infoveillance include examining the cognitive, emotional, and societal factors that make individuals susceptible to sleep-related misinformation, including the role of cognitive biases, social identity, and trust in shaping information consumption and sharing behaviors.

Finally, researchers could innovate and refine metrics and analytical methodologies for tracking, quantifying, and interpreting data on sleep-related information and misinformation, leveraging advances in data science, AI, machine learning, and natural language processing.

This comprehensive research agenda aims not only to illuminate the dynamics of sleep-related information, misinformation, and their consequences but also to develop evidence-based strategies to enhance the public’s ability to navigate the complex information landscape critically and make informed decisions about their sleep health (Table 1).

**Table 1:** Overview of the research agenda of sleep infodemiology and infoveillance.

Research agenda items	Brief description
Map the landscape of sleep-related misinformation	Identify and catalog sleep-related information across platforms
Understand the drivers of sleep-related information-seeking behaviors and their impact on individual (clinical), occupational, and public health	Assess how sleep-related information affects personal health, work performance, and public health
Construct behavioral models from a socioecological perspective	Study the drivers of sleep-related seeking behaviors and the reasons behind the belief and spread of sleep misinformation, including cognitive biases and cultural factors
Explore the role of digital platforms, develop, and evaluate sleep health–related information interventions	Analyze how digital platforms can contribute to the dissemination of high-quality sleep-related information as well as to the spread of sleep-related misinformation
Identify gaps and risk factors in sleep health–related communication	Create and test strategies to correct sleep-related misinformation and promote accurate information
Advance methods in sleep infodemiology and infoveillance	Improve tools and methods for disseminating high-quality sleep-related information, detecting, analyzing, and addressing sleep misinformation

## Conclusion

The complex relationship between sleep-related information, misinformation, and sleep health underscores the need for concerted efforts to combat sleep myths and promote evidence-based sleep practices. Clinical public health initiatives should prioritize sleep education and literacy to empower individuals with accurate information and critical thinking skills necessary to navigate the complex landscape of sleep health–related information. Furthermore, research into the cognitive effects of sleep deprivation should inform strategies to enhance information resilience in society, ensuring that individuals are not only well-rested but also well-equipped to discern truth in an era of pervasive misinformation.

## Abbreviations

AI: artificial intelligence
